# Particle swarm optimization using multi-information characteristics of all personal-best information

**DOI:** 10.1186/s40064-016-3244-8

**Published:** 2016-09-21

**Authors:** Song Huang, Na Tian, Yan Wang, Zhicheng Ji

**Affiliations:** 1 School of Internet of Things Engineering, Jiangnan University, 1800 Lihu Avenue, Wuxi, 214122 Jiangsu Province China; 2 Engineering Research Center of Internet of Things Technology Applications Ministry of Education, Jiangnan University, Wuxi, 214122 China; 3 Department of Educational Technology, Jiangnan University, 1800 Lihu Avenue, Wuxi, Jiangsu Province 214122 China

**Keywords:** Premature convergence, Intelligence algorithm, Particle swarm optimization, Personal-best position

## Abstract

Convergence stagnation is the chief difficulty to solve hard optimization problems for most particle swarm optimization variants. To address this issue, a novel particle swarm optimization using multi-information characteristics of all personal-best information is developed in our research. In the modified algorithm, two positions are defined by personal-best positions and an improved cognition term with three positions of all personal-best information is used in velocity update equation to enhance the search capability. This strategy could make particles fly to a better direction by discovering useful information from all the personal-best positions. The validity of the proposed algorithm is assessed on twenty benchmark problems including unimodal, multimodal, rotated and shifted functions, and the results are compared with that obtained by some published variants of particle swarm optimization in the literature. Computational results demonstrate that the proposed algorithm finds several global optimum and high-quality solutions in most case with a fast convergence speed.

## Background

Particle swarm optimization (PSO) is a bio-inspired optimization algorithm introduced by Eberhart and Kennedy (1995), and is enlightened by the interaction and communication of bird flocking or fish schooling. PSO has attracted a great deal of attention as a treatment for high-dimensional nonlinear optimization problem due to its better computational efficiency and simple implementation. With the development of intelligent manufacturing and complex system, many engineering problems are becoming increasingly complex to optimize, and thus time-consuming computation and premature convergence often occurs in complicated optimization process. Therefore, many PSO variants with new techniques have been proposed to address the above problems.

Some researchers got insight into three control parameters, named after acceleration coefficients and inertia weight, to develop PSO variants (Beielstein et al. 2002; Zhang et al. 2014; Shi and Eberhart 1998a, b). In (Shi and Eberhart 1998b), linearly decreasing inertia weight particle swarm optimization (LPSO) was developed by modified inertia weight and the introduction of this dynamic inertia weight highly strengthened the performance of PSO algorithm. In recent research, multiple swarms or multiple layers strategies had already been proved to be an effective strategy to improve the performance of PSO (Sun and Li 2014; Yadav and Deep 2014; Lim and Isa 2014a; Wang et al. 2014). Sun and Li presented a cooperative particle swarm optimization (TCPSO) with two-swarm (the slave swarm and the master swarm) for optimization problem in large scale search space (Sun and Li 2014) and two subswarms using shrinking hypersphere PSO (SHPSO) and DE were also used in new co-swarm PSO for constrained optimization problems (Yadav and Deep 2014). Multiple layers strategies, such as adaptive two-layer particle swarm optimization algorithm with elitist learning strategy (ATLPSO-ELS) (Lim and Isa 2014a) and multi-layer particle swarm optimization (MLPSO) (Wang et al. 2014), were also used to solve complex problems. PSO with different topologies has different exploration/exploitation ability and performance (Bonyadi et al. 2014; Lim and Isa 2014b, c). Many new topology strategies [time-adaptive topology (Bonyadi et al. 2014), adaptive time-varying topology connectivity (Lim and Isa 2014b), increasing topology connectivity (Lim and Isa 2014c)] were also applied to PSO. Comparing with fully-connected topology or regular topology, these topologies could lead to a different optimization process. In recent years, new techniques such as Levy flight (Haklı and Uğuz 2014), parallel cell coordinate system (Hu and Yen 2015), competitive and cooperative (Li et al. 2015) and orthogonal design (Qin et al. 2015) had also been adopted in PSO.

Many learning strategies are introduced to PSO to enhance the adaptability for complex optimization problems as learning behavior stemming from social animals plays a key role in animals’ adaptation to the changing environment (Cheng and Jin 2015; Rao and Patel 2013; Lim and Isa 2014d; e; Shi and Eberhart 1999). Cheng and Jin presented a modified particle swarm optimization using social learning mechanism (SL-PSO) (Cheng and Jin 2015) and some concept of teachers, tutorial training and self motivated learning was proposed in teaching–learning-based PSO by Rao and Patel for performance enhancements (Rao and Patel 2013). Using teaching and peer-learning behaviors, a bidirectional teaching and peer-learning PSO (BTPLPSO) (Lim and Isa 2014d) and a two learning phases PSO (TPLPSO) (Lim and Isa 2014e) were proposed by Lim and Isa simultaneously.

Communication and learning behavior is a distinguishing feature among social animals and it improves social efficiency. Sharing information mechanism plays a key role in this behavior. To share personal-best information fairness, a particle swarm optimizer using several multi-information characteristics of all personal-best information is developed in this paper. In the proposed PSO, two representative positions, which represent the features of all personal-best positions, are defined to acquiring the information of all personal-best positions. Then the cognition term in velocity update equation is formed by three positions. Due to the effect of all personal-best fitnesses, each particle can update its velocity and position by the distribution of personal-best fitnesses. This strategy could make full use of all personal-best information and correct some error guided directions of personal-best positions.

The structure of rest paper is as follows. Section “Particle swarm optimizer” presents the theory and formulation of PSO algorithm and linearly decreasing inertia weight. In section “Particle swarm optimization using all personal-best information”, the details of two representative positions are described and the proposed PSO using several multi-information characteristics of all personal-best positions is provided. Numerical results and statistical analysis are shown in section “Experiments and results”. In section “Conclusions”, we conclude this paper.

## Particle swarm optimizer

### Velocity and position formulation

Particle swarm optimizer is inspired by fish’s and birds’ foraging behaviors, which are simplified as a swarm of particles by mimicking their key behaviors. As a swarm of *n* particles search in the feasible space, each particle’s position represents a potential solution for the optimization problem and the swarm can find high-quality solution though the particles update their velocities and positions. Assuming the decision has *m* variables, the position and velocity of particle *i* are represented by m-dimensional vector $\varvec{x}_{i} = (x_{i1} ,x_{i2} , \ldots ,x_{{i{\text{m}}}} )$ and $\varvec{v}_{i} = (v_{i1} ,v_{i2} , \ldots ,v_{{i{\text{m}}}} )$. Two positions, named personal-best position and global-best position, are defined in PSO to update the velocities and guide the swarm. Personal-best position of particle *i* is denoted as $\varvec{p}_{{{\text{best}},i}} = (p_{{{\text{best}},i1}} ,p_{{{\text{best}},i2}} , \ldots ,p_{{{\text{best}},i{\text{m}}}} )$ and global-best position of particle *i* is denoted as $\varvec{g}_{\text{best}} = (g_{{{\text{best}},1}} ,g_{{{\text{best}},2}} , \ldots ,g_{{{\text{best}},{\text{m}}}} )$. To sum up, the formulations of the velocity $\varvec{v}_{i}^{t + 1}$ and the position $\varvec{x}_{i}^{t + 1}$ of particle *i* can be expressed by the Eq. (1) and (2).

1 $$\varvec{v}_{i}^{t + 1} = \omega \varvec{v}_{i}^{t} + c_{1} r_{1} \left( {\varvec{p}_{{{\text{best}},i}}^{t} - \varvec{x}_{i}^{t} } \right) + c_{2} r_{2} \left( {\varvec{g}_{\text{best}}^{t} - \varvec{x}_{i}^{t} } \right)$$

2 $$\varvec{x}_{i}^{t + 1} = \varvec{x}_{i}^{t} + \varvec{v}_{i}^{t + 1}$$

where *c*_1_, *c*_2_ are cognitive factor and social factor. *ω* is inertia weight. *r*_1_, *r*_2_ are two real numbers randomly in (0, 1). *t* is the current generation. According to the theory of PSO, the personal experience and global experience make the particle move closer to them to get a new promising position.

### Linearly decreasing inertia weight

Appropriate selection of inertia weight can balance global exploration and local exploitation during the evolution process. Large *ω* can benefit the global search while small value can contribute to local exploitation. Linearly decreasing inertia weight adopted in PSO (LPSO) significantly improves the performance of PSO for solving various optimization problems and the inertia weight *ω* is advised by the Eq. (3):

3 $$\omega ={\upomega}_{\rm max } - ({\upomega}_{\rm max } -{\upomega}_{\rm min } )\frac{t}{T}$$

where ${\text{T}}$ is the maximal generation. ${\upomega}_{\rm{min} }$ and ${\upomega}_{\rm max }$ are the upper limit and lower limit. Numerical experiments illustrated the impact of *ω*, and 0.9 (upper value) and 0.4 (lower value) are suggested (Shi and Eberhart 1999).

From the above description, LPSO pseudo-code is shown in Algorithm 1.
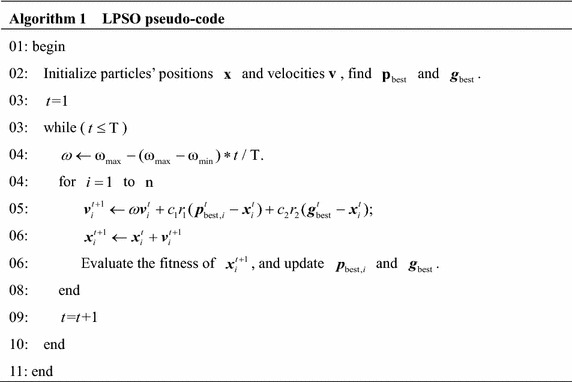


## Particle swarm optimization using all personal-best information

### Analysis of personal-best information

Learning behavior is a special skill for social animals, which can share the information with their members. Cooperative behavior of a swarm is more efficient than one taking an action alone due to their fruitful information and communication. In PSO, each particle can provide its personal-best position information to guide its flying direction. The whole personal-best positions of the swarm imply the distribution of fruitful good-fitness-related information. To take full advantage of multi-information characteristics of all personal-best information will contribute to ignoring several particles’ error information trapping in local optima. In the theory of PSO, personal-best position is only used for its own particle in evolutionary process, not reflecting the influence of fitness distribution in landscape. Misguided information of personal-best positions, which have no opportunities to be corrected, will make PSO premature. Therefore, two positions, which add the influence of personal-best fitness distribution, are defined to strengthen the particle’s ability to learn from other particles’ experience. Then cognition term with three defined personal-best positions in velocity update equation is formed to reduce the misguided opportunity. The details of the improved cognition term and the proposed PSO algorithm are as follows.

### Detail of improved PSO algorithm

*Step 1* Calculate all personal-best positions’ fitnesses, and then figure out the minimal fitness and the maximal fitness among these personal-best fitnesses. The way to find the minimal fitness and the maximal fitness is as follows:

4 $$f_{\rm min } = \hbox{min} \{ f(\varvec{p}_{{{\text{best}},i}} )|i = 1,2, \ldots ,{\text{n}}\}$$

5 $$f_{\rm max } = \hbox{max} \{ f(\varvec{p}_{{{\text{best}},i}} ) |i = 1,2, \ldots ,{\text{n}}\}$$

where *f*_min_ and *f*_max_ stand for the minimal fitness and the maximal fitness of personal-best positions. *f* denotes the fitness function.

*Step 2* Normalization method of personal-best fitness.

As the fitness value varies with a wide range in various optimization problems, a robust way to suitably reflect the influence of fitness is to normalize personal-best fitness. For minimization problem, the smaller the fitness value, the stronger the influence of personal-best position. According this feature, the normalization method is as Eq. (6).

6 $$r_{i} = \frac{{f_{\rm max } - f(\varvec{p}_{{{\text{best}},i}} )}}{{f_{\rm max } - f_{\rm min } }}$$

where *r*_*i*_ stands for the normalized value of the *ith* personal-best fitness.

*Step 3* After normalized the personal-best fitness, we should also acquire the proportions of these fitnesses. The proportion of the *ith* personal-best fitness is denoted as *θ*_*i*_. Thus, for the normalized value of the *ith* personal-best fitness, *θ*_*i*_ can be obtained as follows:

$$\theta_{i} = \left\{ {\begin{array}{*{20}l} {{{r_{i} } \mathord{\left/ {\vphantom {{r_{i} } {\sum\nolimits_{i = 1}^{n} {r_{i} } }}} \right. \kern-0pt} {\sum\nolimits_{i = 1}^{n} {r_{i} } }}} &\quad {{\text{if }}f_{\rm max } \ne f_{\rm min } } \\ {{1 \mathord{\left/ {\vphantom {1 n}} \right. \kern-0pt} n}} &\quad {\text{otherwise}} \\ \end{array} } \right.$$

*Step 4* Calculate centroid position $\varvec{p}_{\text{centr}}$ of all personal-best positions:

7 $$p_{{{\text{centr}},j}} = \sum\limits_{i = 1}^{n} {p_{{{\text{best}},ij}} } \theta_{i}$$

Centroid position is defined as weighted sum form of ${\mathbf{p}}_{\text{best}}$ with $\varvec{\theta}$ to reflect the influence of personal-best fitnesses. Similar to the relation of an object's density and mass in physics, by regarding personal-best fitness as ‘the density of an object’ and personal-best position as ‘the location in the object’, the position $\varvec{p}_{\text{centr}}$ can be seen as ‘the centroid of the object’. The centroid of an object is important factor to reflect the distribution of mass and thus $\varvec{p}_{\text{centr}}$ reflects the distribution of high quality fitness. The centroid position is always close to the area where most good fitnesses locate.

*Step 5* Calculate median position $\varvec{p}_{\text{med}}$ of all personal-best positions.

$\varvec{p}_{\text{med}}$ represents the position of the median personal-best fitness. $\varvec{p}_{\text{med}}$ also reflects the distribution of high quality fitness from another perspective. $\varvec{p}_{\text{med}}$ is obtained without weighted sum form and can avoid the influence of some bad personal-best positions. Algorithm 2 is Pseudo-code to find the median fitness $\theta_{\text{med}}$ and the median position $\varvec{p}_{\text{med}}$.
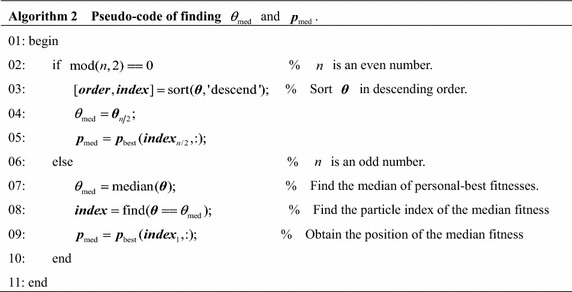


*Step 6* Cognitive guiding position ${\mathbf{p}}_{\text{best}}^{\prime }$.

In the proposed PSO, cognitive guiding position using the above defined positions is calculated according to the following equations:

8 $$\varvec{p}_{{{\text{best}},i}}^{\prime } = \frac{{\varvec{p}_{{{\text{best}},i}} + \varvec{p}_{\text{centr}} - \varvec{p}_{\text{med}} }}{2}$$

The cognitive guiding position includes three positions, the personal-best position ${\mathbf{p}}_{\text{best}}$, the centroid position $\varvec{p}_{\text{centr}}$ and the median position $\varvec{p}_{\text{med}}$. ${\mathbf{p}}_{\text{best}}$ and $\varvec{p}_{\text{centr}} - \varvec{p}_{\text{med}}$ are used to ‘pull’ the particle to escape local optimum because some error information of ${\mathbf{p}}_{\text{best}}$ and $\varvec{g}_{\text{best}}$ may accelerate premature convergence. $\varvec{p}_{\text{centr}}$ and $\varvec{p}_{\text{med}}$ carry all personal-best information and can guide particles to a better direction. The experimental coefficient of 1/2 makes the cognitive guiding position suitable for the improved cognition term.

*Step 7* Improved cognition term ${\mathbf{a}}_{\text{cog}}$.

9 $$\varvec{a}_{{{\text{cog}},i}} = \sum\limits_{i = 1}^{n} {\varvec{p}_{{{\text{best}},i}}^{\prime } \theta_{i} } - \varvec{x}_{i}$$

The improved cognition term ${\mathbf{a}}_{\text{cog}}$ will make full use of all personal-best fitnesses.

*Step 8* Modified velocity update equation.

In this step, the work is to replace original cognition term with the improved cognition term ${\mathbf{a}}_{\text{cog}}$ in velocity update equation of PSO and LPSO algorithm. Therefore, particle swarm optimizer using multi-information characteristics of all personal-best information (PSO-API) and Linearly decreasing inertia weight PSO-API (LPSO-API) can be obtained using this modified velocity update equation. Take LPSO-API algorithm for example, each particle’s velocity updates as Eq. (10).

10 $$\varvec{v}_{i}^{t + 1} = \omega \varvec{v}_{i}^{t} + r_{1} \cdot \varvec{a}_{{{\text{cog}},i}}^{t} + c \cdot r_{2} \cdot \left( {\varvec{g}_{\text{best}}^{t} - \varvec{x}_{i}^{t} } \right)$$

**Fig. 1:**
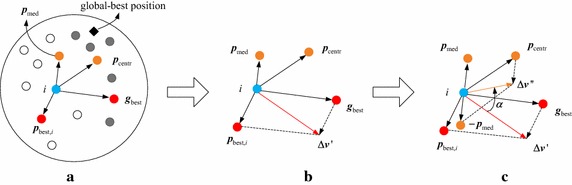
Influence of $\varvec{p}_{\text{centr}}$ and $\varvec{p}_{\text{med}}$ in search process. **a** Distribution of *p*_centr_ and *p*_med_, **b** Influence of all defined best positions, **c** Adjustment of each velocity influenced by all best positions

**Fig. 2:**
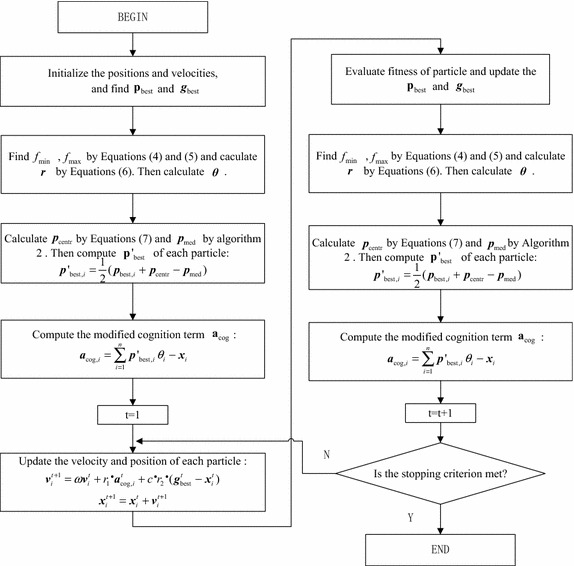
Flowchart of LPSO-API algorithm

Not considering the influence of the current velocity and all the coefficients, there are four positions (${\mathbf{p}}_{\text{best}}$,$\varvec{g}_{\text{best}}$,$\varvec{p}_{\text{centr}}$ and $\varvec{p}_{\text{med}}$) to influence the velocity update in Eq. (10). In PSO, $\Delta \varvec{v}^{\prime } = \varvec{g}_{\text{best}} + \varvec{p}_{{{\text{best}},i}}$ is introduced to show the influence of $\varvec{g}_{\text{best}}$ and $\varvec{p}_{{{\text{best}},i}}$. As is illustrated in Fig. 1b, if the current iteration $\varvec{g}_{\text{best}}$ is local optimum, $\Delta \varvec{v}^{\prime }$ will accelerate the particles fall into local optimum region. Comparing with PSO, $\varvec{p}_{\text{centr}}$ and $\varvec{p}_{\text{med}}$ is added to velocity update equation in PSO-API. In Fig. 1a, the white circle points represents the personal-best positions with worse fitnesses and the grey circle points represents the personal-best positions with better fitnesses. From the distribution of these above points, the location of $\varvec{p}_{\text{centr}}$ and $\varvec{p}_{\text{med}}$, which are calculated by Eq. (7) and Algorithm 2, are shown by the yellow circle points in Fig. 1. Defined by all personal-best positions and their fitnesses, $\varvec{p}_{\text{centr}}$ is more close to the region which locates many personal-best positions with good fitnesses. Although the fitness around the real global-best position is worse than that of local optimum $\varvec{g}_{\text{best}}$, the personal-best positions are also prone to distribute in these positions with good fitnesses around real global-best position, which is the black rhombic point in Fig. 1a. Regard $\varvec{p}_{\text{centr}}$ as a reference point, and $\Delta \varvec{v}^{\prime \prime } = \varvec{p}_{{{\text{best}},i}} - \varvec{p}_{\text{med}}$, which carrys all personal-best information, represents the influence of good fitness distribution. As is illustrated in Fig. 1c, *α* represents the direction adjusted by $\Delta \varvec{v}^{\prime \prime }$ and $\Delta \varvec{v}^{\prime \prime }$ makes particles adjust their directions to the real global-best position. Constantly adjusted by *α* in the search process, the particles have a greater probability of flying to the real global-best position. Besides, $|\Delta \varvec{v}^{\prime \prime } |$ will be small value when an uniform fitness distribution occurs in the search process and $|\Delta \varvec{v}^{\prime \prime } |$ makes little effect on particles. That is, PSO-API only has ${\mathbf{p}}_{\text{best}}$ and $\varvec{g}_{\text{best}}$ influence particles’ trajectory and PSO-API has the same performance as PSO in that case. Therefore, three terms ($\Delta \varvec{v}^{\prime \prime }$,${\mathbf{p}}_{\text{best}}$ and $\varvec{g}_{\text{best}}$) contribute to adjusting the velocity and different ‘pull’ and ‘push’ influence make PSO-API have a stable performance over a variety of problems. The flowchart of LPSO-API algorithm is shown in Fig. 2.

## Experiments and results

### Test benchmark functions

In order to assess the performance of the proposed algorithm, twenty benchmark problems including unimodal, multimodal, rotated and shifted functions selected from the literature (Deep and Thakur 2007; Liang et al. 2006; Suganthan et al. 2005; Yao et al. 1999) are used to verify it. Note that all the problems are minimum problems and only one global optimum exists. The function name, dimensions, search range and global optimum value are listed in Table 1 and the formulations of these problems are listed as follows:

**Table 1:** Twenty benchmark problems

Function name	Dimensions	Search range	Global optimum
*f*_1_(*x*)	20/30/50	[−100, 100]^D^	0
*f*_2_(*x*)	20/30/50	[−10, 10]^D^	0
*f*_3_(*x*)	20/30/50	[−100, 100]^D^	0
*f*_4_(*x*)	20/30/50	[−100, 100]^D^	0
*f*_5_(*x*)	20/30/50	[−100, 100]^D^	0
*f*_6_(*x*)	20/30/50	[−1.28, 1.28]^D^	0
*f*_7_(*x*)	20/30/50	[−5.12, 5.12]^D^	0
*f*_8_(*x*)	20/30/50	[−5.12, 5.12]^D^	0
*f*_9_(*x*)	20/30/50	[−32, 32]^D^	0
*f*_10_(*x*)	20/30/50	[−600, 600]^D^	0
*f*_11_(*x*)	20/30/50	[−0.5, 0.5]^D^	0
*f*_12_(*x*)	20/30/50	[−50, 50]^D^	0
*f*_13_(*x*)	20/30/50	[−1, 1]^D^	0
*f*_14_(*x*)	20/30/50	[−5.12, 5.12]^D^	0
*f*_15_(*x*)	20/30/50	[−100, 100]^D^	0
*f*_16_(*x*)	20/30/50	[−100, 100]^D^	0
*f*_17_(*x*)	20/30/50	[−1.28, 1.28]^D^	0
*f*_18_(*x*)	20/30/50	[−100, 100]^D^	−450
*f*_19_(*x*)	20/30/50	[−32, 32]^D^	−140
*f*_20_(*x*)	20/30/50	[−0.5, 0.5]^D^	90

1. Sphere Function (unimodal function)
  $$f_{1} (x) = \sum\limits_{i = 1}^{n} {x_{i}^{2} }$$
2. Schewefel’s Problem 2.22 (unimodal function)
  $$f_{2} (x) = \sum\limits_{i = 1}^{n} {\left| {x_{i} } \right| + \mathop \prod \limits_{i = 1}^{n} \left| {x_{i} } \right|}$$
3. Schewefel’s Problem 1.2 (unimodal function)
  $$f_{3} (x) = \sum\limits_{i = 1}^{n} {\left( {\sum\limits_{j = 1}^{i} {x_{j} } } \right)^{2} }$$
4. Schewefel’s Problem 2.21 (unimodal function)
  $$f_{4} (x) = \mathop {\hbox{max} }\limits_{i} \{ \left. {\left| {x_{i} } \right|,1 \le i \le n} \right\}$$
5. Step Function (unimodal function)
  $$f_{5} (x) = \sum\limits_{i = 1}^{n} {\left( {\left| {x_{i} + 0.5} \right|} \right)}^{2}$$
6. Quartic Function, i.e. Noise (unimodal function)
  $$f_{6} (x) = \sum\limits_{i = 1}^{n} {ix_{i}^{4} } + random[0,1)$$
7. Generalized Rastrigin’s Function (multimodal function)
  $$f_{7} (x) = \sum\limits_{i = 1}^{n} {\left[ {x_{i}^{2} - 10\cos (2\pi x_{i} ) + 10} \right]}$$
8. Non-continuous Rastrigin’s Function (multimodal function)
  $$\begin{aligned} f_{8} (x) = \sum\limits_{i = 1}^{n} {\left[ {y_{i}^{2} - 10\cos (2\pi y_{i} ) + 10} \right]} \hfill \\ {\text{where}}\quad {\kern 1pt} y_{i} = \left\{ {\begin{array}{*{20}c} {x_{i} } \\ {\frac{{round(2x_{i} )}}{2}} \\ \end{array} } \right.\quad {\kern 1pt} \begin{array}{*{20}c} {\left| {x_{i} } \right| \le 0.5} \\ {\left| {x_{i} } \right| \ge 0.5} \\ \end{array} \hfill \\ \end{aligned}$$
9. Ackley’s Function (multimodal function)
  $$f_{9} (x) = - 20\exp \left( { - 0.2\sqrt {\frac{1}{n}\sum\limits_{i = 1}^{n} {x_{i}^{2} } } } \right) - \exp \left( {\frac{1}{n}\sum\limits_{i = 1}^{n} {\cos 2\pi x_{i} } } \right) + 20 + e$$
10. Generalized Griewank Function (multimodal function)
  $$f_{10} (x) = \frac{1}{4000}\sum\limits_{i = 1}^{n} {x_{i}^{2} } - \prod\limits_{i = 1}^{n} {\cos \left(\frac{{x_{i} }}{\sqrt i }\right)} + 1$$
11. Weierstrass Function (multimodal function)
  $$\begin{aligned} & f_{11} (x) = \sum\limits_{i = 1}^{n} {\left( {\sum\limits_{K = 0}^{k\rm{max} } {\left[ {a^{k} \cos \left( {2\pi b^{k} \left( {x_{i} + 0.5} \right)} \right)} \right]} } \right) - n} \sum\limits_{K = 0}^{k\rm{max} } {\left[ {a^{k} \cos \left( {2\pi b^{k} \times 0.5} \right)} \right]} \hfill \\&\quad {\text{where}}\quad {\kern 1pt} a = 0.5,\quad b = 3,\quad k\hbox{max} = 20 \hfill \\ \end{aligned}$$
12. Generalized Penalized Function (multimodal function)
  $$\begin{aligned}& f_{12} (x) = \tfrac{\pi }{n}\left\{ {10\sin \left( {\pi y_{1} } \right) + \sum\limits_{i = 1}^{n - 1} {\left( {y_{i} - 1} \right)^{2} \left[ {1 + 10\sin^{2} \left( {\pi y_{i + 1} } \right)} \right] + \left( {y_{n} - 1} \right)^{2} } } \right\} + \sum\limits_{i = 1}^{n} u (x_{i} ,10,100,4) \hfill \\&\quad y_{i} = 1 + \frac{{x_{i} + 1}}{4},u(x_{i} ,a,k,m) = \left\{ {\begin{array}{*{20}l} {k\left( {x_{i} - a} \right)^{m} } \\ 0 \\ {k\left( { - x_{i} - a} \right)^{m} } \\ \end{array} } \right.\begin{array}{*{20}c} {x_{i} > a} \\ { - a \le x_{i} \le a} \\ {x_{i} < a} \\ \end{array} \hfill \\ \end{aligned}$$
13. Cosine mixture Problem (multimodal function)
  $$f_{13} (x) = \sum\limits_{i = 1}^{n} {x_{i}^{2} } - 0.1\sum\limits_{i = 1}^{n} {\cos \left( {5\pi x_{i} } \right)}$$
14. Rotated Rastrign Function (multimodal function)
  $$f_{14} (x) = \sum\limits_{i = 1}^{n} {\left[ {y_{i}^{2} - 10\cos (2\pi y_{i} ) + 10} \right], \quad y = M \times x}$$
15. Rotated Salomon Function (multimodal function)
  $$f_{15} (x) = 1 - \cos \left( {2\pi \sqrt {\sum\limits_{i = 1}^{n} {y_{i}^{2} } } } \right) + 0.1\sqrt {\sum\limits_{i = 1}^{n} {y_{i}^{2} } }, \quad y = M \times x$$
16. Rotated Rosenbrock Function (multimodal function)
  $$f_{16} (x) = \sum\limits_{i = 1}^{n - 1} {\left[ {100\left( {y_{i}^{2} - y_{i + 1} } \right)^{2} + \left( {y_{i} - 1} \right)^{2} } \right], \quad y = M \times x}$$
17. Rotated Elliptic Function (unimodal function)
  $$f_{17} (x) = \sum\limits_{i = 1}^{n} {\left( {10^{6} } \right)^{{{{\left( {i - 1} \right)} \mathord{\left/ {\vphantom {{\left( {i - 1} \right)} {\left( {n - 1} \right)}}} \right. \kern-0pt} {\left( {n - 1} \right)}}}} y_{i}^{2}, \quad y = M \times x}$$
18. Shifted Schewefel’s Problem 2.21 (unimodal function)
  $$\begin{aligned} &f_{18} (x) = \mathop {\hbox{max} }\limits_{i} \left\{ {\left| {y_{i} } \right|,1 \le i \le n} \right\} + fbias_{18}, \quad y = x - o \hfill \\&\quad {\text{where}}\quad {\kern 1pt} fbias_{18} = - 450. \hfill \\ \end{aligned}$$
19. Shifted Rotated Ackley’s Function (multimodal function)
  $$\begin{aligned} & f_{19} (x) = - 20\exp \left( { - 0.2\sqrt {\frac{1}{n}\sum\limits_{i = 1}^{n} {z_{i}^{2} } } } \right) - \exp \left( {\frac{1}{n}\sum\limits_{i = 1}^{n} {\cos 2\pi z_{i} } } \right) + 20 + e + fbias_{19} \hfill \\& \quad{\text{where}}\quad {\kern 1pt} fbias_{19} = - 140, \quad z = \left( {x - o} \right) \times M^{\prime } \hfill \\ \end{aligned}$$
20. Shifted Rotated Weierstrass Function (multimodal function)
  $$\begin{aligned} &f_{20} (x) = \sum\limits_{i = 1}^{n} {\left( {\sum\limits_{K = 0}^{k\hbox{max} } {\left[ {a^{k} \cos \left( {2\pi b^{k} \left( {z_{i} + 0.5} \right)} \right)} \right]} } \right) - n} \sum\limits_{K = 0}^{k\hbox{max} } {\left[ {a^{k} \cos \left( {2\pi b^{k} \times 0.5} \right)} \right]} + fbias_{20} \hfill \\&\quad {\text{where}}\quad {\kern 1pt} a = 0.5,\quad b = 3,\quad k\hbox{max} = 20,\quad fbias_{20} = 90,\quad z = \left( {x - o} \right) \times M^{\prime } \hfill \\ \end{aligned}$$

### Experimental analysis

#### Validity of the proposed strategy

To validate the proposed strategy, PSO-API and LPSO-API algorithms are implemented on matlab 2011a to compare with PSO and LPSO algorithms. All twenty benchmarks are tested in the experiments. Parameter settings of the four algorithms are as follows: The size of the population is 30. *c*_1_ and *c*_2_ are both equal to 2 in PSO and LPSO algorithms, and *c* is equal to 2 in PSO-API and LPSO-API algorithms. *ω* is equal to 0.7 in PSO and PSO-API algorithms and uses the suggested linearly decreasing version of section “Linearly decreasing inertia weight” in LPSO and LPSO-API algorithms (Shi and Eberhart 1999). 20, 30 and 50 dimensions are adopted in our experiments and the generations are 5000. Also, 20 independent trials are implemented on these problems. Tables 7, 9 and 11 in “Appendix” show the comparisons of 20, 30, 50 dimensions’ results of average best fitness(AVE), rank(Rank) of average best fitness, median best fitness (MED), standard deviation (SD), average rank (AR) and final rank (FR) of average best fitness.

Wilcoxon’s rank sum test is commonly used to analyze whether two data sets are statistically different from each other, and $p{\text{ value}}$(*p*), ${\text{h-value}}$(h) and ${\text{zval}}$(z) are acquired in Wilcoxon’s rank sum test. In this test, significance level needs to be set and a value of 0.05 significance level indicates that something occurs more than the probability of 95 %. In Wilcoxon’s rank sum test, ${\text{h-value}}$ only has three value, 1, 0, −1, which indicate that the proposed algorithm have a significantly better, same and worse performance than the compared algorithm, respectively (Beheshti et al. 2013). Tables 8, 10 and 12 in “Appendix” show the comparisons of 20, 30, 50 dimensions’ results of Wilcoxon’s rank sum test. In details, the last three rows of Tables 8, 10 and 12 list the numbers of 1, 0 or −1 that ${\text{h-value}}$ equals. Note that the best results for each benchmark function are marked in bold in Tables 7–12.

**Fig. 3:**
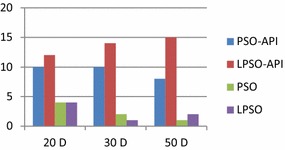
Number of best AVE obtained by four algorithms

From 20, 30 and 50 dimensions’ results in Tables 7, 9 and 11, it is clearly that LPSO-API algorithm obtains the minimum value in terms of AVE on twelve, fourteen, and fifteen of twenty benchmark problems, respectively and PSO-API algorithm obtains the minimum value of AVE on ten, ten and eight of twenty benchmark problems, respectively. It is obviously that LPSO-API algorithm and PSO-API algorithm obtains more minimum results than LPSO algorithm and PSO algorithm in terms of AVE on the suite of benchmark problems. It is worth pointing out that several global optimums are also obtained by LPSO-API algorithm and PSO-API algorithm. The numbers of best AVE obtained by four algorithms are described in Fig. 3.

For three different dimensions, final rank obtained by LPSO-API algorithm all takes the first place and that obtained by PSO-API algorithm are all the second. The final rank can reflect the comprehensive performance of algorithm on a suite of benchmark problems. From the rank, it is clearly seen that LPSO-API algorithm and PSO-API algorithm shows the superiority than LPSO algorithm and PSO algorithm in high-quality solutions.

From the data in Tables 8, 10 and 12, the number of ${\text{h-value = 1}}$ is 16, 17 and 17 for PSO-API algorithm and 13, 14 and 17 for LPSO-API algorithm on 20, 30 and 50 dimensions’ problems. A few ${\text{h-value = }} - 1$ and ${\text{h-value = }}0$ exist. It means that the results of LPSO-API and PSO-API algorithm statistically significantly outperform that of the PSO and LPSO algorithm. Also, by comparing the number of ${\text{h-value = 1}}$ with 20, 30, 50 dimensions’ problems, we can seen that the higher the dimension, the larger the number of ${\text{h-value = 1}}$ of LPSO-API algorithm and PSO-API algorithm. It illustrates that the LPSO-API algorithm and PSO-API algorithm perform better on high-dimension problem than low-dimension problem to some degree. From the above analysis, the proposed strategy of using all personal-best information is valid and efficient for solving most optimization problems, especially in high dimensions.

**Fig. 4:**
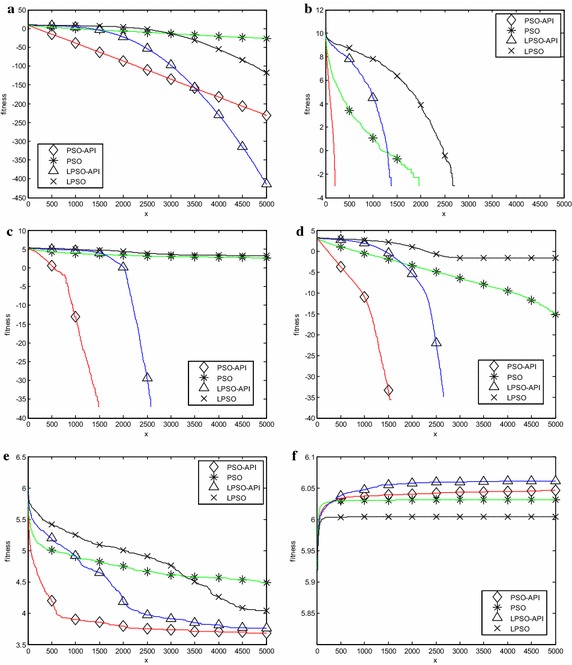
Evolution curves (20 dimensions). **a** $\, f_{1} (x)$, **b** $\, f_{5} (x)$, **c** $\, f_{7} (x)$, **d** $\, f_{11} (x)$, **e** *f*_14_(*x*), **f** *f*_18_(*x*)

**Fig. 5:**
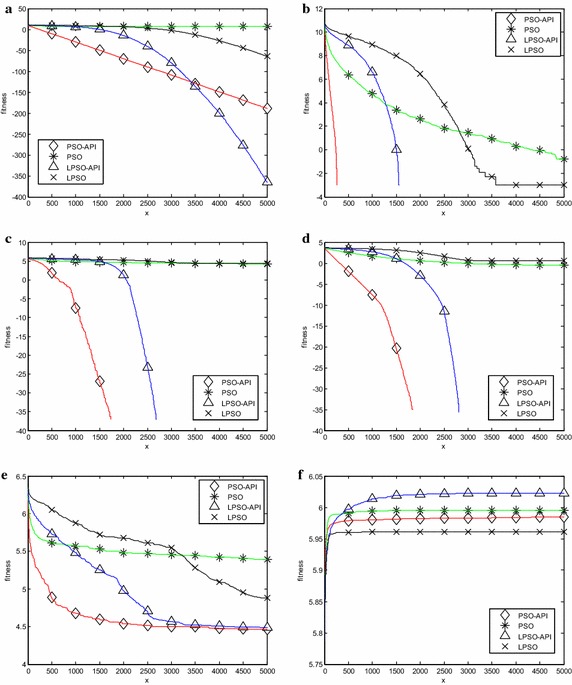
Evolution curves (30 dimensions). **a** $\, f_{1} (x)$, **b** $\, f_{5} (x)$, **c** $\, f_{7} (x)$, **d** $\, f_{11} (x)$, **e** *f*_14_(*x*), **f** *f*_18_(*x*)

**Fig. 6:**
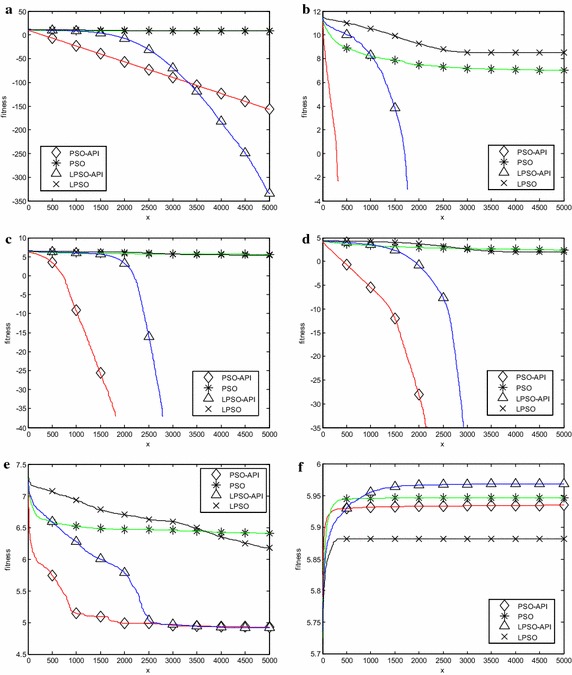
Evolution curves (50 dimensions). **a** $\, f_{1} (x)$, **b** $\, f_{5} (x)$, **c** $\, f_{7} (x)$, **d** $\, f_{11} (x)$, **e** *f*_14_(*x*), **f** *f*_18_(*x*)

Six representative benchmark problems, two unimodal problems $\, f_{1} (x)$ and *f*_5_(*x*), two multimodal problems *f*_7_(*x*) and $f_{ 1 1} (x)$, a rotated problems $f_{ 1 4} (x)$, a shifted problems $f_{ 1 8} (x)$ are chosen for describing the process of fitness evolution. The evolutions of average fitness on these six problems are shown in Figs. 4a–f, 5a–f, 6a–f, respectively. Note that it is the logarithm of average fitness on vertical axis. It is clearly seen from these figures that PSO-API algorithm and LPSO-API algorithm obtain better solution with a fast convergence speed.

#### Comparison experiments with other PSO variants

In recent literatures, various PSO algorithms are also developed and perform well on numerical experiments. To compare with these PSO algorithms, eight PSO variants (PSO-cf (Kennedy and Mendes 2002), FIPS (Mendes et al. 2004), HPSO-TVAC (Ratnaweera et al. 2004), VPSO (Kennedy and Mendes 2006), DMS-PSO (Liang and Suganthan 2005), CLPSO (Liang et al. 2006) and APSO (Zhan et al. 2009) are introduced to optimize ten benchmark functions, which are *f*_1_(*x*), *f*_2_(*x*), *f*_3_(*x*), *f*_5_(*x*), *f*_6_(*x*), *f*_7_(*x*), *f*_8_(*x*), *f*_9_(*x*), *f*_10_(*x*) and *f*_12_(*x*) in section “Test benchmark functions”. Table 2 shows their parameters settings and their results are from the corresponding paper (Zhan et al. 2009). The generations are 2 × 10^5^ and dimension number is 30. The size of population is 20. All the problems are optimized 30 times. Parameters settings of PSO-API and other settings are identical to that in last section. The comparisons of these PSO algorithms are shown in Table 3 in terms of average best fitness (Best) and standard deviation (SD), rank (Rank), average rank (AR) and final rank (FR) of average best fitness. Note that the best results for each benchmark function are marked in bold in Table 3.

**Table 2:** Parameters settings of PSO variants

PSO variant	Topology	Parameters settings
PSO-cf	Local ring	*ω*:0.9 − 0.4, *c*_1_ = *c*_2_ = 2.0
FIPS	Local ring	*χ* = 0.729, ∑ *c*_*i*_ = 4.1
HPSO-TVAC	Global star	*ω*:0.9 − 0.4, *c*_1_:2.5 − 0.5, *c*_2_:0.5 − 2.5
DMS-PSO	Dynamic multi-swarm	*ω*:0.9 − 0.4, *m* = 3, *R* = 5
VPSO	Local von neumann	*ω*:0.9 − 0.4, *c*_1_ = *c*_2_ = 2.0
CLPSO	Comprehensive learning	*ω*:0.9 − 0.4, *C* = 1.49455, *m* = 7
APSO	Global star	$\omega :0.9,c_{1} = c_{2} = 2.0,\delta :{\text{random in [0}} . 0 5 { 0} . 1 ] , { }\sigma : 1 { - 0} . 1$

**Table 3:** Numerical results for the comparisons

Name		PSO-cf	FIPS	HPSO-TVAC	DMS-PSO	VPSO	CLPSO	APSO	PSO-API
$\, f_{1} (x)$	Best	4.77e−29	3.21e−30	3.38e−41	3.85e−54	5.11e−38	1.89e−19	1.45e−150	**0.00**
	SD	1.13e−28	1.91e−30	8.50e−41	1.75e−53	1.91e−37	1.49e−19	5.73e−150	**0.00**
	Rank	7	6	4	3	5	8	2	**1**
$\, f_{2} (x)$	Best	2.03e−20	1.32e−17	6.9e−23	2.61e−29	6.29e−27	1.01e−13	5.15e−84	**3.95e**−**323**
	SD	2.89e−20	7.86e−18	6.89e−23	6.6e−29	8.68e−27	6.51e−14	1.44e−83	**5.13e**−**322**
	Rank	6	7	5	3	4	8	2	**1**
$\, f_{3} (x)$	Best	18.60	0.77	2.89e−7	47.5	1.44	395	1.0e−10	**0.00**
	SD	30.71	0.86	2.97e−7	56.4	1.55	142	2.13e−10	**0.00**
	Rank	6	4	3	7	5	8	2	**1**
$\, f_{5} (x)$	Best	**0.00**	**0.00**	**0.00**	**0.00**	**0.00**	**0.00**	**0.00**	**0.00**
	SD	**0.00**	**0.00**	**0.00**	**0.00**	**0.00**	**0.00**	**0.00**	**0.00**
	Rank	**1**	**1**	**1**	**1**	**1**	**1**	**1**	**1**
$\, f_{6} (x)$	Best	1.49e−2	**2.55e**−**3**	5.54e−2	1.1e−2	1.08e−2	3.92e−3	4.66e−3	5.88e−001
	SD	5.66e−3	**6.25e**−**4**	2.08e−2	3.94e−3	3.24e−3	1.14e−3	1.7e−3	2.73e−001
	Rank	6	**1**	7	5	4	2	3	8
$\, f_{7} (x)$	Best	34.90	29.98	2.39	28.1	34.09	2.57e−11	5.8e−15	**0.00**
	SD	7.25	10.92	3.71	6.42	8.07	6.64e–11	1.01e−14	**0.00**
	Rank	8	6	4	5	7	3	2	**1**
$\, f_{8} (x)$	Best	30.40	21.33	35.91	1.83	32.8	0.167	4.14e−16	**0.00**
	SD	9.23	9.46	9.49	2.65	6.49	0.397	1.45e−15	**0.00**
	Rank	6	5	8	4	7	3	2	**1**
$\, f_{9} (x)$	Best	1.85e−14	7.69e−15	2.06e−10	8.52e−15	1.14e−14	2.01e−12	1.11e−14	**3.55e**−**015**
	SD	4.80e−15	9.33e−16	9.45e−10	1.79e−15	3.48e−15	9.22e−13	3.55e−15	**0.00e+000**
	Rank	6	2	8	3	5	7	4	**1**
$\, f_{10} (x)$	Best	1.10e−2	9.04e−4	1.07e−2	1.31e−2	1.31e−2	6.45e−13	1.67e−2	**0.00**
	SD	1.60e−2	2.78e−3	1.14e−2	1.73e−2	1.35e−2	2.07e−12	2.41e−2	**0.00**
	Rank	5	3	4	7	6	2	8	**1**
$\, f_{12} (x)$	Best	2.18e−30	1.22e−31	7.07e−30	**2.05e**−**32**	3.46e−3	1.59e−21	3.76e−31	9.72e−002
	SD	5.14e−30	4.85e−32	4.05e−30	**8.12e**−**33**	1.89e−2	1.93e−21	1.2e−30	1.97e−002
	Rank	4	2	5	**1**	7	6	3	8
	AR	5.5	3.7	4.9	3.9	5.1	4.8	2.9	**2.4**
	FR	8	3	6	4	7	5	2	**1**

From Table 3, the data of Rank demonstrates that PSO-API algorithm obtains best results on *f*_1_(*x*), *f*_2_(*x*), *f*_3_(*x*), *f*_5_(*x*), *f*_7_(*x*), *f*_8_(*x*), *f*_9_(*x*), *f*_10_(*x*) and performs worst on *f*_6_(*x*) and *f*_12_(*x*). Table 3 also shows FR obtained by PSO-API algorithms is better than that obtained by other eight PSO variants. It can be concluded that PSO-API algorithm has the highest comprehensive performance among them. Consequently, the comparisons indicate that PSO-API algorithm has the best overall performance over several existing PSO variants and is an effective method for solving a variety of optimization problems.

Time complexity of algorithm also should be considered and a computational experiment of six PSO variants [PSO-cf (Kennedy and Mendes 2002), FIPS (Mendes et al. 2004), DMS-PSO (Liang and Suganthan 2005), CLPSO (Liang et al. 2006), LPSO (Shi and Eberhart 1998c) and PSO-API] is performed over 20 independent runs and the execution times of these algorithms are compared. In the experiment, parameters sittings of these algorithms are the same as Table 2. The population, dimension and generations are 20, 30 and 3000, respectively. Table 4 lists CPU times (in seconds) of six PSO algorithms. In Table 4, ‘AV(CPU)’ and ‘Rank’ stand for the average CPU time over 20 runs and the ascending order of each ‘AV(CPU)’, respectively. ‘AR’ and ‘FR’ stand for the average rank of Rank and the ascending order of AR, respectively.

**Table 4:** Computational time of six PSO algorithms

Function		PSO-cf	FIPS	DMS-PSO	CLPSO	LPSO	PSO-API
$\, f_{1} (x)$	AV(CPU)/Rank	6.09e−001/1	4.19e+000/6	4.04e+000/5	3.60e+000/4	7.59e−001/2	1.28e+000/3
$\, f_{2} (x)$	AV(CPU)/Rank	1.76e+000/3	4.10e+000/5	4.85e+000/6	3.55e+000/4	9.99e−001/1	1.51e+000/2
$\, f_{3} (x)$	AV(CPU)/Rank	1.01e+001/2	1.16e+001/3	1.28e+001/4	9.95e+000/1	1.44e+001/5	1.61e+001/6
$\, f_{4} (x)$	AV(CPU)/Rank	3.06e+000/3	4.19e+000/5	5.50e+000/6	3.67e+000/4	1.13e+000/1	1.66e+000/2
$\, f_{5} (x)$	AV(CPU)/Rank	3.31e+000/3	4.12e+000/6	4.11e+000/5	3.78e+000/4	8.22e−001/1	1.27e+000/2
$\, f_{6} (x)$	AV(CPU)/Rank	5.78e+000/1	7.04e+000/4	8.51e+000/6	6.79e+000/3	6.51e+000/2	7.35e+000/5
$\, f_{7} (x)$	AV(CPU)/Rank	3.17e+000/3	4.49e+000/5	4.56e+000/6	3.98e+000/4	1.03e+000/1	1.46e+000/2
$\, f_{8} (x)$	AV(CPU)/Rank	5.05e+000/2	6.59e+000/5	6.96e+000/6	5.89e+000/4	4.72e+000/1	5.31e+000/3
$\, f_{9} (x)$	AV(CPU)/Rank	4.83e+000/3	6.31e+000/5	7.23e+000/6	5.66e+000/4	3.18e+000/1	3.89e+000/2
$\, f_{10} (x)$	AV(CPU)/Rank	4.21e+000/1	6.53e+000/5	6.83e+000/6	6.16e+000/4	4.74e+000/2	5.20e+000/3
$\, f_{11} (x)$	AV(CPU)/Rank	5.09e+001/2	5.17e+001/3	6.52e+001/4	4.78e+001/1	9.52e+001/5	9.80e+001/6
$\, f_{12} (x)$	AV(CPU)/Rank	6.19e+000/1	1.22e+001/3	1.25e+001/4	1.12e+001/2	1.80e+001/6	1.75e+001/5
$\, f_{13} (x)$	AV(CPU)/Rank	4.52e−002/1	3.98e+000/6	3.91e+000/5	3.51e+000/4	1.07e+000/2	1.46e+000/3
$\, f_{14} (x)$	AV(CPU)/Rank	3.66e+000/3	4.78e+000/5	4.97e+000/6	4.24e+000/4	2.51e+000/1	2.94e+000/2
$\, f_{15} (x)$	AV(CPU)/Rank	3.81e+000/3	4.90e+000/5	5.00e+000/6	4.47e+000/4	2.72e+000/1	3.15e+000/2
$\, f_{16} (x)$	AV(CPU)/Rank	4.49e+000/2	5.57e+000/5	6.62e+000/6	5.10e+000/4	4.14e+000/1	4.51e+000/3
$\, f_{17} (x)$	AV(CPU)/Rank	4.80e+000/3	5.90e+000/5	1.02e+001/6	4.67e+000/1	4.79e+000/2	5.35e+000/4
$\, f_{18} (x)$	AV(CPU)/Rank	3.12e−003/1	4.60e+000/5	5.70e+000/6	3.67e+000/4	2.19e+000/2	2.58e+000/3
$\, f_{19} (x)$	AV(CPU)/Rank	1.56e−003/1	5.84e+000/5	6.25e+000/6	4.35e+000/3	4.13e+000/2	4.57e+000/4
$\, f_{20} (x)$	AV(CPU)/Rank	2.65e+001/2	2.76e+001/3	3.25e+001/4	2.00e+001/1	4.99e+001/6	4.95e+001/5
	AR	2.05	4.7	5.45	3.2	2.25	3.35
	FR	1	5	6	3	2	4

From Rank of LPSO and PSO-API algorithm, we can conclude that our proposed policy adding to the original PSO increases the computational time. In Table 4, AR reflects comprehensive time-consuming order of the algorithm for twenty benchmarks. From Table 4, the value of AR for PSO-cf and LPSO are smallest among all six algorithms. It illustrates that PSO-cf and LPSO, which are better than our proposed algorithm, have the best CPU time. The value of ‘AR’ for PSO-API algorithm and CLPSO are highly close to each other and it demonstrates that they have similar overall time consumption. The value of ‘AR’ for PSO-cf and LPSO are ‘4.7’ and ‘5.45’, which are both worse than PSO-API algorithm. From the value of ‘FR’, although PSO-API algorithm only ranks four, it is worthy of spending time to improve the accuracy of PSO algorithm. It is clear from the above comparisons of the accuracy and time consumption that PSO-API algorithm has a good overall balance between the performance and time complexity.

#### Comparisons experiments with similar PSO algorithms

In order to compare with FSS (Carmelo Filho et al. 2008) and CenterPSO (Liu et al. 2007), several experiments are carried out in this section. To compare with FSS algorithm, the experiments settings are as follows: five benchmarks with 30 dimensions are used to assess the algorithms. In detail, Generalized Rosenbrock Function and *f*_3_(*x*), *f*_7_(*x*), *f*_9_(*x*), *f*_10_(*x*) in section “Test benchmark functions” are introduced. Generalized Rosenbrock Function is denoted as *f*_21_(*x*), and the expression of *f*_21_(*x*) is shown as follows.

$$f_{21} (x) = \sum\limits_{i = 1}^{n - 1} {(100(x_{i + 1} - x_{i}^{2} )^{2} + (x_{i} - 1)^{2} )} \quad { (} - 1 0 0\le x_{i} \le 100 )$$

Population size of PSO-API sets as 30. 30 runs are conducted for each problem and each run will perform 1 × 10^4^ generations. To compare with CenterPSO algorithm, the experiments settings are as follows: Three benchmarks *f*_7_(*x*), *f*_10_(*x*), *f*_21_(*x*) with 30 dimensions are used. The generations is 2000. The population has four sizes of 20, 40, 80, 160. Each experiment will perform 100 runs. Average best fitness (Avg. best fitness) and standard deviation (SD) of PSO-API, FSS and CenterPSO are presented in Tables 5 and 6. It’s worth noting that the better results are marked in bold in Tables 5 and 6.

**Table 5:** Comparison results with FSS algorithm

Name		PSO-API	FSS
$\, f_{3} (x)$	Avg. best fitness/SD	**3.883e−090/9.563e−090**	8.080e−002/2.200e−002
$\, f_{7} (x)$	Avg. best fitness/SD	**0.000e+000/0.000e+000**	1.338e+001/4.005e+000
$\, f_{9} (x)$	Avg. best fitness/SD	**3.789e**−**015/9.013e**−**016**	4.000e−002/2.000e−002
$\, f_{10} (x)$	Avg. best fitness/SD	**0.000e+000/0.000e+000**	2.700e−003/2.000e−003
$\, f_{21} (x)$	Avg. best fitness/SD	2.635e+001/**3.145e**−**001**	**1.611e+001/**7.290e−001

**Table 6:** Comparison results with CenterPSO algorithm

Name	Size		PSO-API	CenterPSO
$\, f_{7} (x)$	20	Avg. best fitness/SD	**0.000e+000/0.000e+000**	3.359e+001/9.562e+000
	40	Avg. best fitness/SD	**0.000e+000/0.000e+000**	2.668e+001/7.764e+000
	80	Avg. best fitness/SD	**0.000e+000/0.000e+000**	2.276e+001/6.758e+000
	160	Avg. best fitness/SD	**2.020e−010/2.020e−009**	2.141e+001/5.949e+000
$\, f_{10} (x)$	20	Avg. best fitness/SD	**2.311e−004/1.711e−003**	1.200e−002/1.650e−002
	40	Avg. best fitness/SD	**7.841e−005/7.841e−004**	8.800e−003/1.190e−002
	80	Avg. best fitness/SD	**8.442e−006/8.442e−005**	9.300e−003/1.200e−002
	160	Avg. best fitness/SD	**7.308e**−**015/7.151e**−**014**	1.200e−002/1.680e−002
$\, f_{21} (x)$	20	Avg. best fitness/SD	**2.702e+001/3.831e**−**001**	1.319e+002/1.358e+002
	40	Avg. best fitness/SD	**2.649e+001/3.276e**−**001**	8.717e+001/6.365e+001
	80	Avg. best fitness/SD	**2.626e+001/1.987e**−**001**	6.234e+001/5.940e+001
	160	Avg. best fitness/SD	**2.601e+001/2.379e**−**001**	4.299e+001/4.499e+001

From the data in Table 5, it can be seen that PSO-API obtains better average best fitness and standard deviation than that obtained by FSS algorithm for all five benchmarks except Generalized Rosenbrock Function. However, for Generalized Rosenbrock Function, PSO-API and FSS algorithm obtain the results with the same order of magnitude. From the Table 6, the results obtained by PSO-API with all population sizes are better than that obtained by CenterPSO algorithm for all three benchmarks. Therefore, statistics analysis indicates the proposed algorithm have better performance than FSS algorithm and CenterPSO algorithm. For most of the benchmarks, all above experiments indicates that PSO-API is a high-performance algorithm.

## Conclusions

In this work, to make full use of multi-information characteristics of all personal-best information, an improved PSO algorithm using three positions with all personal-best information has been adopted to enhance the performance. In proposed algorithm, an improved cognition term using the personal-best position, the centroid position and the median position is introduced in velocity update process of PSO. To validate this strategy, a set of benchmark functions including unimodal, multimodal, rotated and shifted benchmark functions with 20, 30 and 50 dimensions have been optimized. Experimental results show that the strategy using multi-information characteristics of all personal-best information is a valid strategy for the purposes of improving the PSO’s performance. Moreover, PSO-API algorithm has also been used to compare with several PSO variants and some similar algorithms of the proposed algorithm. Numerical results show that the PSO-API algorithm has higher precision and satisfied performance. To sum up, the proposed strategy enhances the search ability of PSO and PSO-API algorithm is an efficient PSO variant to obtain promising solution for most of benchmark functions.

## Appendix

See Tables 7, 8, 9, 10, 11, 12.

**Table 7:** Results of twenty benchmark problems (generations = 5000 and dimensions = 20)

Name		PSO-API	PSO	LPSO-API	LPSO
*f*_1_(*x*)	AVE/rank	6.143e−101/2	3.714e−012/4	**1.992e**−**180/1**	5.879e−052/3
	MED/SD	2.803e−102/1.498e−100	6.501e−013/8.397e−012	**4.447e**−**183/2.124e**−**180**	1.476e−053/1.638e−051
*f*_2_(*x*)	AVE/rank	1.522e−050/2	1.000e+000/3	**3.973e**−**091/1**	3.000e+000/4
	MED/SD	7.388e−051/2.773e−050	1.387e−010/3.077e+000	**7.565e**−**092/1.174e**−**090**	2.895e−033/4.701e+000
*f*_3_(*x*)	AVE/rank	1.049e−060/2	3.145e+003/3	**3.938e**−**132/1**	8.000e+003/4
	MED/SD	8.599e−062/3.394e−060	2.604e+003/3.448e+003	**5.529e**−**134/1.488e**−**131**	1.000e+004/5.342e+003
*f*_4_(*x*)	AVE/rank	8.837e−042/2	2.666e+000/4	**1.290e**−**078/1**	5.100e−004/3
	MED/SD	1.414e−042/1.569e−041	2.376e+000/1.136e+000	**1.071e**−**079/4.198e**−**078**	3.224e−004/6.771e−004
*f*_5_(*x*)	AVE/rank	**0.0e+000/1**	**0.0e+000/1**	**0.0e+000/1**	**0.0e+000/1**
	MED/SD	**0.0e+000/0.0e+000**	**0.0e+000/0.0e+000**	**0.0e+000/0.0e+000**	**0.0e+000/0.0e+000**
*f*_6_(*x*)	AVE/rank	4.992e−001/3	7.748e−001/4	4.983e−001/2	**4.625e**−**001/1**
	MED/SD	5.276e−001/3.032e−001	8.512e−001/3.869e−001	**5.067e**−**001**/2.680e−001	5.071e−001**/2.670e**−**001**
*f*_7_(*x*)	AVE/rank	**0.0e+000/1**	1.411e+001/3	**0.0e+000/1**	2.490e+001/4
	MED/SD	**0.0e+000/0.0e+000**	9.455e+00/1.125e+001	**0.0e+00/0.0e+000**	1.542e+01/1.996e+001
*f*_8_(*x*)	AVE/rank	**0.0e+000/1**	2.034e+001/3	1.773e+000/2	2.530e+001/4
	MED/SD	**0.0e+000/0.0e+000**	1.867e+01/1.158e+001	**0.0e+00**/6.846e+000	2.700e+01/1.999e+001
*f*_9_(*x*)	AVE/rank	**3.552e**−**015/1**	2.440e−007/3	**3.552e**−**015/1**	7.676e−001/4
	MED/SD	**3.552e**−**015/0.0e+000**	1.615e−007/2.245e−007	**3.552e**−**015/0.0e+000**	7.105e−015/3.432e+000
*f*_10_(*x*)	AVE/rank	**0.0e+000/1**	4.861e−002/4	7.404e−004/2	3.588e−002/3
	MED/SD	**0.0e+000/0.0e+000**	4.785e−002/3.737e−002	**0.0e+000**/3.311e−003	2.834e−002/3.710e−002
*f*_11_(*x*)	AVE/rank	**0.0e+000/1**	2.615e−007/3	**0.0e+000/1**	2.000e−001/4
	MED/SD	**0.0e+000/0.0e+000**	1.650e−008/9.516e−007	**0.0e+000/0.0e+000**	0.000e+000/8.944e−001
*f*_12_(*x*)	AVE/rank	5.335e−002/4	**3.893e**−**008/1**	5.103e−002/3	7.775e−003/2
	MED/SD	5.383e−002/9.641e−003	6.931e−014**/1.741e**−**007**	4.906e−002/1.051e−002	**2.355e**−**032**/3.477e−002
*f*_13_(*x*)	AVE/rank	−**2.0e+000/1**	−**2.0e+000/1**	−**2.0e+000/1**	−**2.0e+000/1**
	MED/SD	−**2.0e+000/0.0e+000**	−**2.0e+000**/3.520e−015	−**2.0e+000/0.0e+000**	−**2.0e+000/0.0e+000**
*f*_14_(*x*)	AVE/rank	**3.963e+001/1**	8.864e+001/4	4.278e+001/2	5.657e+001/3
	MED/SD	**3.702e+01**/1.884e+001	8.617e+01/3.462e+001	4.612e+01**/1.646e+001**	5.323e+01/2.357e+001
*f*_15_(*x*)	AVE/rank	**9.987e**−**002/1**	1.370e+000/4	**9.987e**−**002/1**	1.199e+000/3
	MED/SD	**9.987e**−**002/2.883e**−**010**	4.998e−001/3.682e+000	**9.987e**−**002**/4.206e−010	3.998e−001/3.579e+000
*f*_16_(*x*)	AVE/rank	**1.876e+001/1**	3.382e+007/3	1.877e+001/2	4.691e+008/4
	MED/SD	**1.876e+001**/2.135e−002	1.952e+03/1.374e+008	1.877e+001**/1.374e**−**002**	1.971e+08/5.627e+008
*f*_17_(*x*)	AVE/rank	1.529e−012/2	1.252e+004/4	**8.245e**−**035/1**	8.891e+003/3
	MED/SD	1.549e−032/6.838e−012	4.387e+03/1.629e+004	**8.006e**−**096/3.687e**−**034**	6.856e+03/8.742e+003
*f*_18_(*x*)	AVE/rank	−4.224e+002/2	−4.161e+002/3	−**4.288e+002/1**	−4.051e+002/4
	MED/SD	−4.229e+02/3.728e+000	−4.173e+02/1.819e+001	−**4.290e+02/2.251e+000**	−4.077e+02/1.290e+001
*f*_19_(*x*)	AVE/rank	−1.1921e+2/4	−**1.1931e+2/1**	−1.1925e+2/3	−1.1928e+2/2
	MED/SD	−1.1920e+2/6.117e−002	−1.1928e+2/1.049e−001	−1.1923e+2**/5.970e**−**002**	−**1.1928e+2**/7.102e−002
*f*_20_(*x*)	AVE/rank	1.0953e+002/4	1.047e+002/2	1.0951e+002/3	**1.042e+002/1**
	MED/SD	1.095e+02/2.107e+000	1.050e+02/2.457e+000	1.094e+002**/2.029e+000**	**1.037e+02**/2.714e+000
	AR	1.85	2.9	**1.55**	2.9
	FR	2	3	**1**	3

**Table 8:** Wilcoxon’s rank sum test results (generations = 5000 and dimensions = 20)

Name		PSO-API and PSO	LPSO-API and LPSO
*f*_1_(*x*)	p/h/z	6.795e−008/1/−5.396e+000	6.795e−008/1/−5.396e+000
*f*_2_(*x*)	p/h/z	6.795e−008/1/−5.396e+000	6.690e−008/1/−5.399e+000
*f*_3_(*x*)	p/h/z	6.795e−008/1/−5.396e+000	6.795e−008/1/−5.396e+000
*f*_4_(*x*)	p/h/z	6.795e−008/1/−5.396e+000	6.795e−008/1/−5.396e+000
*f*_5_(*x*)	p/h/z	0.000e+000/0/0.000e+000	0.000e+000/0/0.000e+000
*f*_6_(*x*)	p/h/z	3.151e−002/1/−2.150e+000	7.352e−001/0/3.381e−001
*f*_7_(*x*)	p/h/z	8.006e−009/1/−5.768e+000	8.006e−009/1/−5.768e+000
*f*_8_(*x*)	p/h/z	8.006e−009/1/−5.768e+000	3.519e−007/1/−5.093e+000
*f*_9_(*x*)	p/h/z	8.006e−009/1/−5.768e+000	2.412e−008/1/−5.579e+000
*f*_10_(*x*)	p/h/z	8.006e−009/1/−5.768e+000	2.859e−007/1/−5.132e+000
*f*_11_(*x*)	p/h/z	8.006e−009/1/−5.768e+000	1.625e−001/1/−1.396e+000
*f*_12_(*x*)	p/h/z	6.795e−008/1/5.396e+000	2.776e−007/1/5.137e+000
*f*_13_(*x*)	p/h/z	3.122e−006/1/−4.662e+000	0.000e+000/0/0.000e+000
*f*_14_(*x*)	p/h/z	3.293e−005/1/−4.152e+000	9.090e−002/0/−1.690e+000
*f*_15_(*x*)	p/h/z	6.709e−008/1/−5.398e+000	5.612e−008/1/−5.430e+000
*f*_16_(*x*)	p/h/z	1.200e−006/1/−4.855e+000	6.795e−008/1/−5.396e+000
*f*_17_(*x*)	p/h/z	6.795e−008/1/−5.396e+000	6.795e−008/1/−5.396e+000
*f*_18_(*x*)	p/h/z	1.402e−001/0/−1.474e+000	1.025e−007/1/−5.322e+000
*f*_19_(*x*)	p/h/z	3.381e−004/−1/3.584e+000	1.555e−001/0/1.420e+000
*f*_20_(*x*)	p/h/z	3.499e−006/−1/4.639e+000	2.061e−006/−1/4.747e+000
	1	**16**	**13**
	0	2	6
	−1	2	1

**Table 9:** Results of twenty benchmark problems (generations = 5000 and dimensions = 30)

Name		PSO-API	PSO	LPSO-API	LPSO
*f*_1_(*x*)	AVE/rank	1.499e−082/2	1.500e+003/4	**2.291e**−**159/1**	1.184e−028/3
	MED/SD	9.419e−084/3.947e−082	1.782e−003/3.663e+003	**6.706e**−**163/9.471e**−**159**	2.128e−029/3.017e−028
*f*_2_(*x*)	AVE/rank	4.736e−042/2	5.000e+000/3	**5.600e**−**082/1**	1.450e+001/4
	MED/SD	2.354e−042/6.150e−042	4.860e−004/6.069e+000	**2.822e**−**082/6.334e**−**082**	1.500e+001/9.986e+000
*f*_3_(*x*)	AVE/rank	1.395e−043/2	1.510e+004/3	**2.721e**−**108/1**	1.660e+004/4
	MED/SD	1.715e−044/2.946e−043	1.580e+004/6.208e+003	**1.049e**−**109/5.623e**−**108**	1.666e+004/5.629e+003
*f*_4_(*x*)	AVE/rank	5.252e−032/2	1.769e+001/4	**1.338e**−**066/1**	1.968e+000/3
	MED/SD	1.688e−032/8.290e−032	1.719e+001/3.485e+000	**8.649e**−**068/3.874e**−**066**	1.846e+000/7.950e−001
*f*_5_(*x*)	AVE/rank	**0.000e+000/1**	4.500e−001/4	**0.000e+000/1**	5.000e−002/3
	MED/SD	**0.000e+000/0.000e+000**	**0.000e+000**/8.255e−001	**0.000e+000/0.000e+000**	**0.000e+000**/2.236e−001
*f*_6_(*x*)	AVE/rank	5.199e−001/2	2.045e+000/3	**4.889e**−**001/1**	2.307e+000/4
	MED/SD	5.157e−001**/3.248e**−**001**	1.456e+000/1.958e+000	**4.368e**−**001**/3.293e−001	6.323e−001/4.144e+000
*f*_7_(*x*)	AVE/rank	**0.000e+000/1**	6.980e+001/3	**0.000e+000/1**	7.686e+001/4
	MED/SD	**0.000e+000/0.000e+000**	7.259e+001/2.296e+001	**0.000e+000/0.000e+000**	7.522e+001/2.848e+001
*f*_8_(*x*)	AVE/rank	**0.000e+000/1**	1.018e+002/4	**0.000e+000/1**	8.270e+001/3
	MED/SD	**0.000e+000/0.000e+000**	9.411e+001/3.081e+001	**0.000e+000/0.000e+000**	8.001e+001/3.588e+001
*f*_9_(*x*)	AVE/rank	**3.552e**−**015/1**	7.264e−001/3	**3.552e**−**015/1**	3.807e+000/4
	MED/SD	**3.552e**−**015/0.000e+000**	7.494e−003/3.189e+000	**3.552e**−**015/0.000e+000**	2.131e−014/6.874e+000
*f*_10_(*x*)	AVE/rank	**0.000e+000/1**	5.139e−002/3	3.720e−004/2	1.354e+001/4
	MED/SD	**0.000e+000/0.000e+000**	4.340e−002/5.443e−002	**0.000e+000**/1.664e−003	1.845e−002/3.301e+001
*f*_11_(*x*)	AVE/rank	**0.000e+000/1**	6.248e−001/3	**0.000e+000/1**	1.805e+000/4
	MED/SD	**0.000e+000/0.000e+000**	2.362e−002/1.952e+000	**0.000e+000/0.000e+000**	2.395e−003/3.033e+000
*f*_12_(*x*)	AVE/rank	1.078e−001/3	2.256e−001/4	1.067e−001/2	**5.183e**−**003/1**
	MED/SD	1.040e−001**/1.480e**−**002**	1.098e−002/5.944e−001	1.030e−001/1.504e−002	**1.634e**−**028**/2.318e−002
*f*_13_(*x*)	AVE/rank	−**3.000e+000/1**	−2.999e+000/3	−**3.000e+000/1**	−2.940e+000/4
	MED/SD	−**3.000e+00/0.000e+000**	−2.999e+000/7.685e−006	−**3.000e+00/0.000e+000**	−**3.000e+00**/2.683e−001
*f*_14_(*x*)	AVE/rank	**8.703e+001/1**	2.200e+002/4	8.865e+001/2	1.319e+002/3
	MED/SD	**9.867e+001**/4.002e+001	2.177e+002**/3.608e+001**	1.101e+002/4.941e+001	1.186e+002/6.466e+001
*f*_15_(*x*)	AVE/rank	**9.987e**−**002/1**	2.446e+000/3	**9.987e**−**002/1**	1.025e+001/4
	MED/SD	**9.987e**−**002/5.355e**−**010**	2.350e+000/6.174e−001	**9.987e**−**002**/7.443e−010	1.529e+001/9.398e+000
*f*_16_(*x*)	AVE/rank	**2.869e+001/1**	1.573e+009/3	2.870e+001/2	2.542e+009/4
	MED/SD	**2.869e+001/1.449e**−**002**	1.095e+006/3.957e+009	2.870e+001/1.788e−002	1.082e+009/4.418e+009
*f*_17_(*x*)	AVE/rank	1.786e−036/2	4.287e+003/3	**3.634e**−**104/1**	9.561e+003/4
	MED/SD	1.651e−037/3.264e−036	3.560e+003/2.774e+003	**2.468e**−**110/1.625e**−**103**	7.780e+003/6.709e+003
*f*_18_(*x*)	AVE/rank	−3.972e+002/3	−4.016e+002/2	**-4.127e+002/1**	−3.880e+002/4
	MED/SD	−3.965e+02**/3.643e+000**	−4.060e+02/1.933e+001	−**4.127e+02**/4.270e+000	−3.966e+02/2.133e+001
*f*_19_(*x*)	AVE/rank	−1.190e+002/4	−**1.190e+002/1**	−1.190e+002/3	−1.190e+002/2
	MED/SD	−1.190e+002/5.490e−002	−**1.190e+02/6.539e**−**002**	−1.190e+002/4.999e−002	−1.190e+002/1.005e−001
*f*_20_(*x*)	AVE/rank	1.244e+002/3	**1.153e+002/1**	1.270e+002/4	1.172e+002/2
	MED/SD	1.239e+002/2.857e+000	**1.154e+002**/3.703e+000	1.266e+002**/2.562e+000**	1.166e+002/2.931e+000
	AR	1.75	3.05	**1.45**	3.4
	FR	2	3	**1**	4

**Table 10:** Wilcoxon’s rank sum test results (generations = 5000 and dimensions = 30)

Name		PSO-API and PSO	LPSO-API and LPSO
*f*_1_(*x*)	p/h/z	6.795e−008/1/−5.396e+000	6.795e−008/1/−5.396e+000
*f*_2_(*x*)	p/h/z	6.795e−008/1/−5.396e+000	6.690e−008/1/−5.399e+000
*f*_3_(*x*)	p/h/z	6.795e−008/1/−5.396e+000	6.795e−008/1/−5.396e+000
*f*_4_(*x*)	p/h/z	6.795e−008/1/−5.396e+000	6.795e−008/1/−5.396e+000
*f*_5_(*x*)	p/h/z	9.496e−003/1/−2.593e+000	3.421e−001/0/−9.500e−001
*f*_6_(*x*)	p/h/z	1.575e−006/1/−4.801e+000	3.851e−002/1/−2.069e+000
*f*_7_(*x*)	p/h/z	8.006e−009/1/−5.768e+000	8.006e−009/1/−5.768e+000
*f*_8_(*x*)	p/h/z	8.006e−009/1/−5.768e+000	8.006e−009/1/−5.768e+000
*f*_9_(*x*)	p/h/z	8.006e−009/1/−5.768e+000	6.764e−009/1/−5.796e+000
*f*_10_(*x*)	p/h/z	8.006e−009/1/−5.768e+000	2.208e−006/1/−4.733e+000
*f*_11_(*x*)	p/h/z	8.006e−009/1/−5.768e+000	3.494e−007/1/−5.094e+000
*f*_12_(*x*)	p/h/z	7.711e−003/−1/2.664e+000	3.415e−007/−1/5.098e+000
*f*_13_(*x*)	p/h/z	8.006e−009/1/−5.768e+000	1.625e−001/0/−1.396e+000
*f*_14_(*x*)	p/h/z	6.757e−008/1/−5.397e+000	1.476e−001/0/−1.447e+000
*f*_15_(*x*)	p/h/z	6.776e−008/1/−5.396e+000	6.766e−008/1/−5.397e+000
*f*_16_(*x*)	p/h/z	6.795e−008/1/−5.396e+000	6.795e−008/1/−5.396e+000
*f*_17_(*x*)	p/h/z	6.795e−008/1/−5.396e+000	6.795e−008/1/−5.396e+000
*f*_18_(*x*)	p/h/z	1.545e−002/−1/2.421e+000	8.505e−006/1/−4.452e+000
*f*_19_(*x*)	p/h/z	9.277e−005/1/3.908e+000	6.389e−002/0/1.852e+000`
*f*_20_(*x*)	p/h/z	3.938e−007/−1/5.071e+000	9.172e−008/−1/5.342e+000
	1	**17**	**14**
	0	0	4
	−1	3	2

**Table 11:** Results of twenty benchmark problems (generations = 5000 and dimensions = 50)

Name		PSO-API	PSO	LPSO-API	LPSO
*f*_1_(*x*)	AVE/rank	8.111e−069/2	4.571e+003/4	**6.638e**−**146/1**	3.000e+003/3
	MED/SD	2.816e−069/1.213e−068	1.378e+002/6.841e+003	**4.585e**−**152/2.968e**−**145**	9.040e−012/4.701e+003
*f*_2_(*x*)	AVE/rank	1.339e−035/2	2.635e+001/3	**1.770e**−**076/1**	3.800e+001/4
	MED/SD	6.996e−036/1.559e−035	3.020e+001/1.696e+001	**5.086e**−**078/6.311e**−**076**	3.000e+001/1.735e+001
*f*_3_(*x*)	AVE/rank	7.320e−029/2	6.095e+004/3	**6.921e**−**091/1**	7.035e+004/4
	MED/SD	4.714e−031/3.121e−028	6.084e+004/1.020e+004	**2.327e**−**092/1.678e**−**090**	6.774e+004/2.032e+004
*f*_4_(*x*)	AVE/rank	9.832e−024/2	4.042e+001/4	**1.467e**−**055/1**	2.482e+001/3
	MED/SD	3.494e−024/1.388e−023	4.021e+001/4.275e+000	**1.603e**−**057/5.564e**−**055**	2.621e+001/3.161e+000
*f*_5_(*x*)	AVE/rank	**0.000e+000/1**	1.106e+003/3	**0.000e+000/1**	5.000e+003/4
	MED/SD	**0.000e+000/0.000e+000**	1.130e+002/3.090e+003	**0.000e+000/0.000e+000**	**1.000e+000**/6.069e+003
*f*_6_(*x*)	AVE/rank	**4.829e**−**001/1**	1.498e+001/3	5.144e−001/2	1.795e+001/4
	MED/SD	**4.569e**−**001**/2.745e−001	1.618e+001/9.801e+000	5.114e−001**/2.600e**−**001**	1.068e+001/1.779e+001
*f*_7_(*x*)	AVE/rank	**0.000e+000/1**	2.677e+002/4	**0.000e+000/1**	2.329e+002/3
	MED/SD	**0.000e+000/0.000e+000**	2.671e+002/5.346e+001	**0.000e+000/0.000e+000**	2.276e+002/4.966e+001
*f*_8_(*x*)	AVE/rank	1.259e+001/2	2.729e+002/4	**0.000e+000/1**	2.725e+002/3
	MED/SD	**0.000e+000**/5.634e+001	2.803e+002/5.489e+001	**0.000e+000/0.000e+000**	2.680e+002/5.470e+001
*f*_9_(*x*)	AVE/rank	4.973e−015/2	9.682e+000/3	**4.618e**−**015/1**	1.185e+001/4
	MED/SD	**3.552e**−**015**/1.785e−015	1.316e+001/5.389e+000	**3.552e**−**015/1.670e**−**015**	1.370e+001/7.483e+000
*f*_10_(*x*)	AVE/rank	**0.000e+000/1**	2.885e+001/3	**0.000e+000/1**	3.159e+001/4
	MED/SD	**0.000e+000/0.000e+000**	2.355e+000/4.211e+001	**0.000e+000/0.000e+000**	1.599e−002/5.296e+001
*f*_11_(*x*)	AVE/rank	**0.000e+000/1**	1.107e+001/4	**0.000e+000/1**	7.587e+000/3
	MED/SD	**0.000e+000/0.000e+000**	1.186e+001/3.728e+000	**0.000e+000/0.000e+000**	6.002e+000/4.384e+000
*f*_12_(*x*)	AVE/rank	2.453e−001/3	5.749e+003/4	2.361e−001/2	**9.655e**−**002/1**
	MED/SD	2.475e−001/3.344e−002	1.219e+002/1.672e+004	2.427e−001**/2.283e**−**002**	**6.220e**−**002**/1.673e−001
*f*_13_(*x*)	AVE/rank	−**5.000e+000/1**	−4.573e+000/4	−**5.000e+000/1**	−4.611e+000/3
	MED/SD	−**5.000e+00/0.000e+000**	−4.771e+000/4.670e−001	−**5.000e+00/0.000e+000**	−4.926e+000/8.788e−001
*f*_14_(*x*)	AVE/rank	1.381e+002/2	6.056e+002/4	**1.368e+002/1**	4.808e+002/3
	MED/SD	2.115e+002/1.298e+002	5.709e+002/1.049e+002	**2.102e+002/1.290e+002**	4.337e+002/2.018e+002
*f*_15_(*x*)	AVE/rank	**9.987e**−**002/1**	1.897e+001/3	**9.987e**−**002/1**	2.485e+001/4
	MED/SD	**9.987e**−**002/5.210e**−**010**	1.899e+001/6.756e+000	**9.987e**−**002**/5.908e−010	2.559e+001/7.030e+000
*f*_16_(*x*)	AVE/rank	**4.862e+001/1**	1.186e+010/3	4.865e+001/2	1.631e+010/4
	MED/SD	**4.862e+001/1.551e**−**002**	4.668e+009/1.816e+010	4.864e+001/2.043e−002	1.336e+010/1.745e+010
*f*_17_(*x*)	AVE/rank	6.371e−031/2	8.266e+004/3	**7.198e**−**097/1**	1.111e+005/4
	MED/SD	1.426e−031/1.347e−030	7.732e+004/4.103e+004	**4.644e**−**104/3.219e**−**096**	8.578e+004/8.496e+004
*f*_18_(*x*)	AVE/rank	−3.782e+002/3	−3.824e+002/2	−**3.911e+002/1**	−3.586e+002/4
	MED/SD	−3.788e+002**/1.934e+000**	−3.834e+002/1.983e+001	−**3.909e+002**/3.716e+000	−3.539e+002/1.941e+001
*f*_19_(*x*)	AVE/rank	−1.1883e+002/3	−**1.1890e+002/1**	−1.1882e+002/4	−1.1889e+002/2
	MED/SD	−1.1883e+02**/3.887e**−**002**	−**1.1889e+02**/3.944e−002	−1.1881e+02/4.171e−002	−1.1887e+02/7.086e−002
*f*_20_(*x*)	AVE/rank	1.565e+002/3	1.435e+002/2	1.597e+002/4	**1.426e+002/1**
	MED/SD	1.566e+002**/3.440e+000**	**1.421e+002**/5.957e+000	1.590e+002/3.475e+000	1.436e+002/3.741e+000
	AR	1.8	3.2	**1.4**	3.25
	FR	2	3	**1**	4

**Table 12:** Wilcoxon’s rank sum test results (generations = 5000 and dimensions = 50)

Name		PSO-API and PSO	LPSO-API and LPSO
*f*_1_(*x*)	p/h/z	6.795e−008/1/−5.396e+000	6.786e−008/1/−5.396e+000
*f*_2_(*x*)	p/h/z	6.795e−008/1/−5.396e+000	6.795e−008/1/−5.396e+000
*f*_3_(*x*)	p/h/z	6.795e−008/1/−5.396e+000	6.795e−008/1/−5.396e+000
*f*_4_(*x*)	p/h/z	6.795e−008/1/−5.396e+000	6.795e−008/1/−5.396e+000
*f*_5_(*x*)	p/h/z	8.006e−009/1/−5.768e+000	2.457e−005/1/−4.218e+000
*f*_6_(*x*)	p/h/z	6.795e−008/1/−5.396e+000	6.795e−008/1/−5.396e+000
*f*_7_(*x*)	p/h/z	8.006e−009/1/−5.768e+000	8.006e−009/1/−5.768e+000
*f*_8_(*x*)	p/h/z	3.046e−008/1/−5.538e+000	8.006e−009/1/−5.768e+000
*f*_9_(*x*)	p/h/z	3.959e−008/1/−5.492e+000	3.290e−008/1/−5.525e+000
*f*_10_(*x*)	p/h/z	8.006e−009/1/−5.768e+000	8.006e−009/1/−5.768e+000
*f*_11_(*x*)	p/h/z	8.006e−009/1/−5.768e+000	8.006e−009/1/−5.768e+000
*f*_12_(*x*)	p/h/z	6.795e−008/1/−5.396e+000	4.600e−004/−1/3.502e+000
*f*_13_(*x*)	p/h/z	8.006e−009/1/−5.768e+000	7.991e−009/1/−5.768e+000
*f*_14_(*x*)	p/h/z	5.726e−008/1/−5.427e+000	4.505e−007/1/−5.046e+000
*f*_15_(*x*)	p/h/z	6.795e−008/1/−5.396e+000	6.786e−008/1/−5.396e+000
*f*_16_(*x*)	p/h/z	6.795e−008/1/−5.396e+000	6.795e−008/1/−5.396e+000
*f*_17_(*x*)	p/h/z	6.795e−008/1/−5.396e+000	6.795e−008/1/−5.396e+000
*f*_18_(*x*)	p/h/z	1.055e−002/−1/2.557e+000	1.194e−006/1/−4.856e+000
*f*_19_(*x*)	p/h/z	4.165e−005/−1/4.098e+000	4.600e−004/−1/3.502e+000
*f*_20_(*x*)	p/h/z	3.938e−007/−1/5.071e+000	6.795e−008/−1/5.396e+000
	1	**17**	**17**
	0	0	0
	−1	3	3
